# Personal

**Published:** 1915-04

**Authors:** 


### PERSONAL.

Announcement of removal, travel, and other matters of interest are requested. Please report errors in the listing of any physician in the State and other directories, that we may co-operate with the State Society in securing a correct list.

Dr. W. B. Cochrane, of Rochester, has moved from 571 to 1293 Park Avenue.

Dr. Robert J. Talbot, of Niagara Falls, has recently broken his arm, cranking his automobile. (Note—We happened on a case of this sort last summer, at a time when a starter that we had been using was acting so badly that we had decided to remove it entirely. The lesson was so impressive that we got a starter of another kind. See advertising pages.)

---

Brigadier General Wm. C. Goethals has been made a Major General, retaining his position as Surgeon-General of the Army. This is a much more sensible action than to confuse the organization of the various government medical services by making him a special cabinet officer.

---

Dr. Louis J. Regan, of Buffalo, sustained a bad sprain of the right knee in an automobile collision on February 27.

---

Dr. Warren Britt, of Tonawanda, has been appointed Health Officer and City Physician of Tonawanda.

---

Dr. Herbert Upham Williams, of Buffalo, has moved to 40 Irving Place.

---

Dr. Fred M. Meader, formerly city bacteriologist of Syracuse, has been appointed Superintendent of Sanitary Supervisors of New York State.

---

Misses Nellie E. Bundy and Bessie Scanlon, of the class of 1910 of the Buffalo Hospital of the Sisters of Charity have been appointed to the Red Cross and assigned to unit in charge of Dr. Edward W. Ryan of Scranton, at Belgrade, Servia.

---

Dr. Charles Cary of Buffalo has returned from a three months' visit to France and Belgium.

---

Dr. Fred S. Hoffman, of Buffalo, made a trip to Florida and Havana in March.

---

Dr. J. C. Clemesha, of Buffalo, has returned from Florida.

---

Dr. Edward Meyer, of Buffalo, visited San Francisco and the coast in March.

---

Dr. Elmer Starr, of Buffalo, is visiting in Charleston, South Carolina.

